# Discovery of host-directed modulators of virus infection by probing the SARS-CoV-2–host protein–protein interaction network

**DOI:** 10.1093/bib/bbac456

**Published:** 2022-10-27

**Authors:** Vandana Ravindran, Jessica Wagoner, Paschalis Athanasiadis, Andreas B Den Hartigh, Julia M Sidorova, Aleksandr Ianevski, Susan L Fink, Arnoldo Frigessi, Judith White, Stephen J Polyak, Tero Aittokallio

**Affiliations:** Oslo Centre for Biostatistics and Epidemiology (OCBE), Faculty of Medicine, University of Oslo, Oslo, Norway; Institute for Cancer Research, Department of Cancer Genetics, Oslo University Hospital, Oslo, Norway; Department of Laboratory Medicine & Pathology, University of Washington, Seattle, WA, USA; Oslo Centre for Biostatistics and Epidemiology (OCBE), Faculty of Medicine, University of Oslo, Oslo, Norway; Institute for Cancer Research, Department of Cancer Genetics, Oslo University Hospital, Oslo, Norway; Department of Laboratory Medicine & Pathology, University of Washington, Seattle, WA, USA; Department of Laboratory Medicine & Pathology, University of Washington, Seattle, WA, USA; Institute for Molecular Medicine Finland (FIMM), HiLIFE, University of Helsinki, Helsinki, Finland; Department of Laboratory Medicine & Pathology, University of Washington, Seattle, WA, USA; Oslo Centre for Biostatistics and Epidemiology (OCBE), Faculty of Medicine, University of Oslo, Oslo, Norway; Department of Cell Biology and Department of Microbiology, University of Virginia, Charlottesville, VA, USA; Department of Laboratory Medicine & Pathology, University of Washington, Seattle, WA, USA; Oslo Centre for Biostatistics and Epidemiology (OCBE), Faculty of Medicine, University of Oslo, Oslo, Norway; Institute for Cancer Research, Department of Cancer Genetics, Oslo University Hospital, Oslo, Norway; Institute for Molecular Medicine Finland (FIMM), HiLIFE, University of Helsinki, Helsinki, Finland

**Keywords:** cell context specificity, host modulators, network prioritization, protein–protein interactions, SARS-CoV-2

## Abstract

The ongoing coronavirus disease 2019 (COVID-19) pandemic has highlighted the need to better understand virus–host interactions. We developed a network-based method that expands the severe acute respiratory syndrome coronavirus-2 (SARS-CoV-2)–host protein interaction network and identifies host targets that modulate viral infection. To disrupt the SARS-CoV-2 interactome, we systematically probed for potent compounds that selectively target the identified host proteins with high expression in cells relevant to COVID-19. We experimentally tested seven chemical inhibitors of the identified host proteins for modulation of SARS-CoV-2 infection in human cells that express ACE2 and TMPRSS2. Inhibition of the epigenetic regulators bromodomain-containing protein 4 (BRD4) and histone deacetylase 2 (HDAC2), along with ubiquitin-specific peptidase (USP10), enhanced SARS-CoV-2 infection. Such proviral effect was observed upon treatment with compounds JQ1, vorinostat, romidepsin and spautin-1, when measured by cytopathic effect and validated by viral RNA assays, suggesting that the host proteins HDAC2, BRD4 and USP10 have antiviral functions. We observed marked differences in antiviral effects across cell lines, which may have consequences for identification of selective modulators of viral infection or potential antiviral therapeutics. While network-based approaches enable systematic identification of host targets and selective compounds that may modulate the SARS-CoV-2 interactome, further developments are warranted to increase their accuracy and cell-context specificity.

## Introduction

The global outbreak of severe acute respiratory syndrome coronavirus-2 (SARS-CoV-2) requires the development or re-positioning of effective and safe therapies against the virus and coronavirus disease 2019 (COVID-19). In particular, drug repurposing enables a fast track to identify small molecules that target emergent viruses, including SARS-CoV-2, by focusing on viral targeted host proteins and their cellular pathways as targets for the therapeutic intervention. In response to infection, host cells mount antiviral responses. However, SARS-CoV-2, like many other viruses, has developed various strategies to escape cellular antiviral responses, similarly to dengue, SARS-CoV-1 and MERS-CoV [1–3]. It is therefore important to elucidate the interactions of the SARS-CoV-2 with host proteins to gain better insights into virus infection and pathogenesis, and to potentially reveal novel options for therapeutic intervention.

Recent large-scale proteomic and genomic profiling studies have elucidated various mechanisms by which SARS-CoV-2 alters host cells [4, 5]. Several studies have utilized virus–host protein–protein interactions (PPIs) to construct networks of SARS-CoV-2-induced pathways and to uncover novel targets and potential host-targeting agents [6–8]. Some of these network-based studies have also provided a comprehensive resource of the biological pathways and potential drugs for the SARS-CoV-2 targeted host proteins [9–11]. However, many of the small molecules identified through computational network studies have not been experimentally validated, which hinders the discovery of novel compounds or combinations [12]. For instance, Gysi *et al*. experimentally screened 918 compounds in VeroE6 cells and found that none of the predictive algorithms offers consistently reliable outcomes across all metrics and against investigational compounds in clinical trials [13]. Another challenge in experimental testing of model predictions is the high cell context specificity of the compound responses, often making the screening results based on a single-cell line challenging to reproduce in other relevant cell types.

Here, we developed a computational–experimental approach that integrates virus–host protein interactions with both a human PPI network and tissue expression profiles to (i) prioritize host proteins that can be targeted by compounds in clinical development and (ii) to experimentally probe how these compounds modulate virus infection. The key feature of our integrated approach is that it exploits the cell context-specific dependencies of the virus on specific host proteins and pathways during replication. To expand the host targets, biological pathways and compound spaces, we used a random walk with restart, a probabilistic network propagation algorithm, to identify additional host targets from the extended set of nearest neighbours of virus-interacting host proteins (VIPs). We explored the biological function and targeting effect of such network-inferred proteins (NIPs), which may not interact directly with the viral proteins but can be functionally related to VIPs through various pathways within the SARS-CoV-2 interactome.

We tested selective inhibitors of the identified host proteins in two cell lines (HEK293T cells expressing ACE2 and TMPRSS2 and lung epithelial Calu 3 cells) with multidose assays to understand the dose- and context-specific effects of the inhibitors on viral infection in relevant cell models. Interestingly, we identified several host-targeting compounds that enhance virus infection, suggesting that the target proteins act as antivirals. Furthermore, two host-targeting compounds showed modest suppressive effects on virus infection, and no toxic effects in mock-infected control cells, demonstrating that even though the network-based approach identified therapeutically safe targets, it cannot distinguish between the virus suppressive and enhancing effects. When comparing to other screens of SARS-CoV-2 compounds, we observed cell context-specific differences in antiviral effects across cell models. Collectively, our computational–experimental approach and its findings support the discovery of novel host-targeting modulators of virus infection, as well as of novel chemical tools for probing how virus–host interactions regulate virus infection.

## Results

### Viral interacting proteins are critically positioned for network information flow

**Figure 1:**
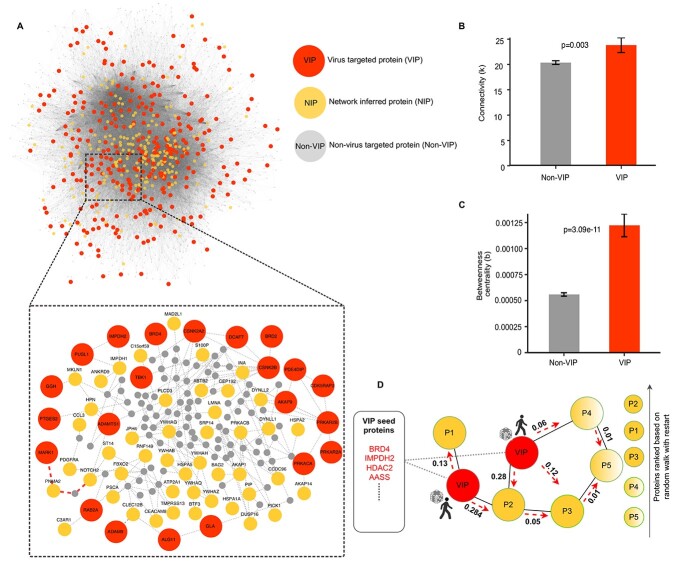
Topological network analysis of the SARS-CoV-2 PPI network. (**A**) The giant component of the SARS-CoV-2–host PPI network that contains 297 VIPs and 3678 non-VIPs. The inset shows a subnetwork of select VIP nodes. When calculating the neighbourhood size from VIP to non-VIP nodes, the connections marked in red show the shortest path distance from the MARK1 VIP node to NOTCH2 node in the network (neighbourhood size of 3). (**B**) Degree, i.e. the number of connections, for VIP and non-VIP nodes (the bars show mean ± SEM). (**C**) Betweenness centrality, i.e. the number of shortest paths through a given node, for VIP and non-VIP nodes (mean ± SEM). (**D**) Operation of the RWR (RWR) algorithm. The red seed nodes are the set of 298 VIPs from which the random walker starts exploring the network (marked by red arrows). After iterating through all nodes in the network, a probability score is assigned to all nodes in the network, ranked from the highest to the lowest probability, which was used to identify 200 top-ranked NIP nodes.

Similar to other viral infections, the SARS-CoV-2 life cycle is mediated through a complex system of protein interactions, modelled here as network graphs, with cellular proteins depicted as nodes and their interactions as edges. To model the system-wide virus–host interactions, we used a PPI network among 298 human proteins identified previously as interacting with SARS-CoV-2 virus proteins. Since the initial characterization of virus-interacting host proteins (VIPs) was carried out in the HEK293T cell line [14], we constructed our SARS-CoV-2 PPI network in this same cellular context by mapping the interactions between the VIPs and their nearest neighbours derived from pull-down experiments reported in the Bioplex interactome [15]. The HEK293T PPI network consists of 3978 protein nodes and 41 015 interaction edges, and it has two connected components: a giant component of 3975 nodes and a smaller component of 3 nodes (Supplementary Table S1a available online at http://bib.oxfordjournals.org/). In the downstream network analyses, we focused on the giant component that contains 297 VIPs and their 3678 nearest neighbour proteins (non-VIPs) that are not targeted directly by the viral proteins (Figure 1A).

To investigate the role of the host proteins targeted by the virus in the PPI network, we calculated several network information measures that quantify the topological inter-connectivity of the giant network component (Supplementary Table S1b available online at http://bib.oxfordjournals.org/). The connectivity (*k*), i.e. the number of direct interacting partners of each protein, showed that the SARS-CoV-2 VIPs have a higher connectivity (mean 23.82), compared to the non-virus-targeted host proteins (non-VIPs) (mean 20.36, *P* = 0.0026, Wilcoxon test; Figure 1B). The central position of VIPs in the network information flow became even more pronounced in terms of the betweenness centrality (*b*), i.e. the number of shortest paths between each pair of nodes that pass-through a given node, which showed that VIPs have significantly higher centrality (mean 0.0012), compared to non-VIPs (mean 0.00055, *P* = 3.084 × 10^−11^, Wilcoxon test; Figure 1C). This observation is consistent with other studies that have shown that viruses tend to target network hubs and bottlenecks in the PPI network [16, 17], since viruses and host proteins are constantly competing for binding partners that interact with proteins in various pathways during the infection phase.

### Identification of additional host proteins that are indirectly regulated by VIPs

To further study the information flow through VIPs, we characterized their neighbourhood sizes, i.e. the length of the shortest paths from a VIP to any non-VIP nodes in the giant component of the network. We found that most VIP nodes can be reached by traversing as few as three or four edges from any other non-VIP node. This is comparable to the average path length from a random node to any other node in the network (mean 3.422, Supplementary Figure S1 available online at http://bib.oxfordjournals.org/). This topological network analysis provides information for the positioning of host-directed modulators of virus infection that are either directly interacting with viral proteins (VIPs) or are components of viral-regulated pathways, where proteins do not directly interact with viral proteins (i.e. non-VIPs) but are nonetheless required for viral replication. In particular, the central position of VIPs in the network makes it critical to investigate the potential side effects of inhibiting these host targets, as network hubs often interact with many other proteins involved in normal cellular processes and biological pathways, targeting of which may lead to toxic effects on the non-infected cell [18].

The above network analysis suggests that it is also important to consider host proteins that are not directly targeted by viral proteins yet are part of pathways regulated by viruses. For such extended host target mapping, we employed random walk with restart (RWR), a probabilistic network propagation algorithm [19], which was applied here in the identification of proteins connected to VIPs (see Supplementary Text available online at http://bib.oxfordjournals.org/). The RWR algorithm explores the network vicinity and function of the protein seed set (here, VIPs), based on the premise that proteins with similar functions tend to lie close to each other in the PPI network. Thus, the RWR algorithm identifies proteins within the network that may be functionally similar to the VIPs by topologically scanning each node in the PPI network from the VIP seed set and then assigns a probability score to the non-seed proteins (Figure 1D). The proteins were ranked based on the probability assigned by the RWR algorithm, so that the higher the probability, the closer the proteins are to the VIPs. In this way, we shortlisted 200 top-ranked proteins and termed them as NIPs (Supplementary Table S1b available online at http://bib.oxfordjournals.org/) (Figure 1A).

### Biological pathway characterization of the identified host targets

**Figure 2:**
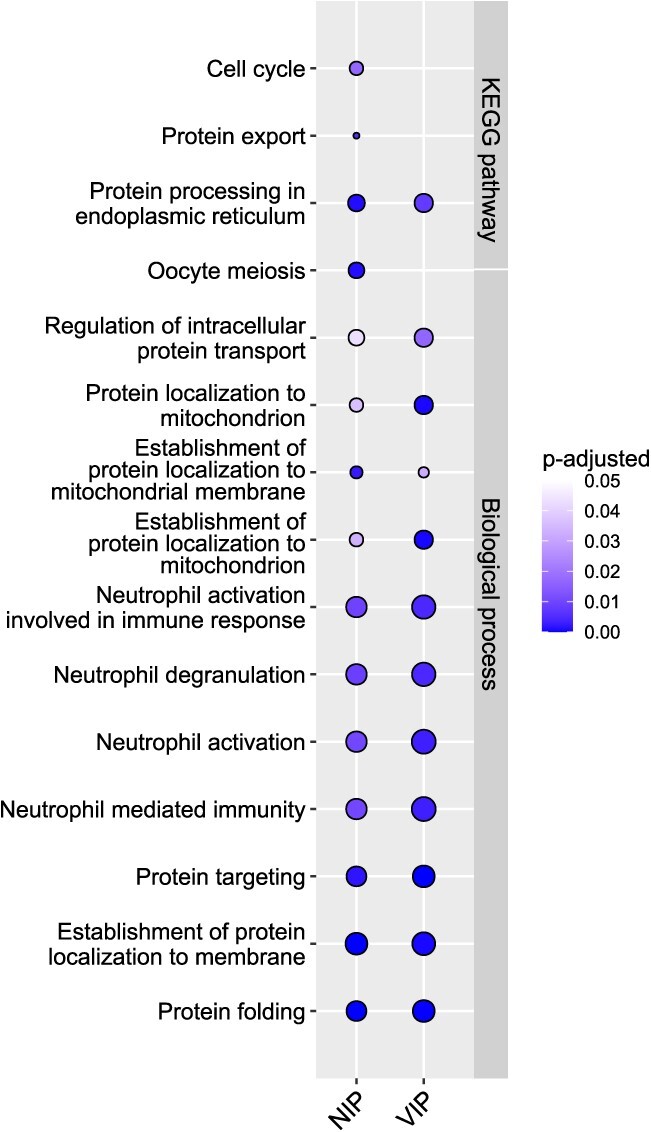
GO biological process and KEGG pathways that are commonly or uniquely enriched among the NIP and VIP targets. The enrichment *P*-values were corrected for multiple testing with Benjamini–Hochberg test, and the pathways with adjusted *P* < 0.05 were considered significant. The dots are colour-coded based on their corresponding adjusted *P*-values and the dot size corresponds to the gene ratio (i.e. genes of interest in the GO term/total number of genes of interest.

To investigate whether the network-inferred proteins (NIPs) also share similar biological function with the VIPs, as suggested by their network similarity, we performed gene set enrichment analysis, separately for the VIP and NIP targets, and then compared the biological pathways and Gene Ontology (GO) terms between the two target sets. A total of 11 biological processes were similarly enriched in the NIP and VIP sets, including protein folding (*P* = 6.70 × 10^−8^, 1.43 × 10^−7^), establishment of protein localization to membranes (P = 6.80 × 10^−8^, 2.90 × 10^−6^) and protein targeting (*P* = 6.59 × 10^−6^, 2.62 × 10^−8^) (Figure 2). Apart from these biological processes that are known to be involved in viral processing [20, 21], we also identified several common biological processes between the two host target sets that are related to immune responses, such as neutrophil activation, mediated immune responses and degranulation (Supplementary Table S2 available online at http://bib.oxfordjournals.org/). We further observed that the network-predicted NIP targets shared other relevant pathways with the VIP set in terms of KEGG pathways. For instance, the NIP and VIP targets were both enriched for proteins involved in processing in the endoplasmic reticulum (ER) pathway (*P* = 1.198 × 10^−5^, 3.72 × 10^−5^; Figure 2), which is biologically plausible as the ER is involved in viral replication and assembly [22].

Interestingly, the NIP target set also extended biological processes beyond those captured by the VIP targets. For instance, NIP targets were enriched for the cell cycle pathway (*P* = 3.62 × 10^−4^), which highlights its importance in viral replication [23, 24]. These network-based results also support previous studies that have shown how SARS-CoV-2 hijacks the rough endoplasmic reticulum (RER)-linked host translational machinery for its replication [11]. In addition, NIP targets were enriched for pathways related to protein export (*P* = 8.99 × 10^−5^), which also includes secretory pathways that are hijacked by the virus to carry out their essential functions, such as virus replication, assembly and egress, demonstrating that viruses evade host cellular pathways to promote their propagation [25]. These results indicate that the extended target set consisting of the top-200 NIPs identified by the RWR algorithm do not only populate similar pathways as VIPs but are also implicated in various biological processes related to viral infection.

### Identification of host-targeted compounds that modulate viral infection

**Figure 3:**
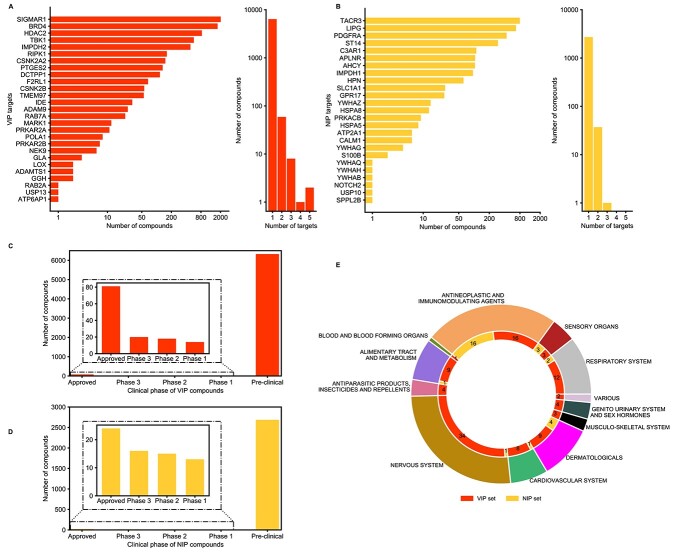
Identification of potent compounds that inhibit the target proteins based on ChEMBL database (bioactivity <1000 nM, see Materials and Methods). (**A**) The number of compounds identified for the 27 VIP targets (left histogram), and the number of VIP targets for each potent compound (right histogram). (**B**) The number of compounds identified for the 25 NIP targets (left), and the number of NIP targets for each potent compound (right). (**C**) Clinical development phase of compounds that target VIPs. (**D**) Clinical development phase of compounds that target NIPs. (**E**) Anatomical Therapeutic Chemical (ATC) classification of the 101 approved compounds that target select VIPs and NIPs.

To disrupt the SARS-CoV-2 interactome, we sought potent and selective compounds that inhibit the identified host proteins (both VIPs and NIPs). We prioritized drugs that are approved for other indications and investigational compounds currently being tested in clinical trials (phase 1–3), instead of preclinical candidates that would take longer time to develop as COVID-19 modulators. We first identified compounds that inhibit the identified host targets (VIPs and NIPs) using target activity data from the ChEMBL database [26]. For the VIP targets, 6458 unique compounds were identified that cover 27 of the 298 targets (9%; Figure 3A). For the NIPs, 2754 unique compounds were identified that cover 25 of the top-200 targets (13%; Figure 3B).

Considering the number of compounds for VIP and NIP targets, many of the identified proteins are targeted by multiple compounds, suggesting that most of these host targets are already well studied in drug discovery (Figure 3A and B, left distributions). The distribution of compounds per target between the VIPs and the NIPs were relatively similar (*P* = 0.75, Kolmogorov–Smirnov test). Notably, most of the compounds have been reported to show activity against a single target only among these target sets, suggesting that the inhibitors may be selective against the host targets in each set (Figure 3A and B, right distributions). However, most of the compounds that selectively target VIPs and NIPs are still in a pre-clinical phase (Figure 3C and D). This systematic compound screen resulted in a total of 9079 potent inhibitors of the 52 host proteins (27 VIPs and 25 NIPs) (Supplementary Table S3 available online at http://bib.oxfordjournals.org/), out of which only 101 (0.01%) are currently approved for other indications (Figure 3E), i.e. representing potential repurposing opportunities.

### Selection of a subset of host targets that is most relevant for COVID-19

To select the host targets and their inhibitory compounds for experimental validation, we further shortlisted our target lists from both the VIP and NIP sets, based on the expression levels of the host proteins in cells relevant for COVID-19 using expression data from the Human Protein Atlas [27]. Specifically, we chose to use the target expression in lung epithelial cells and cells of the upper and lower respiratory tract, since the virus infects the upper respiratory tract, causing flu-like symptoms, and the lower respiratory tract, causing severe respiratory disorders.

**Figure 4:**
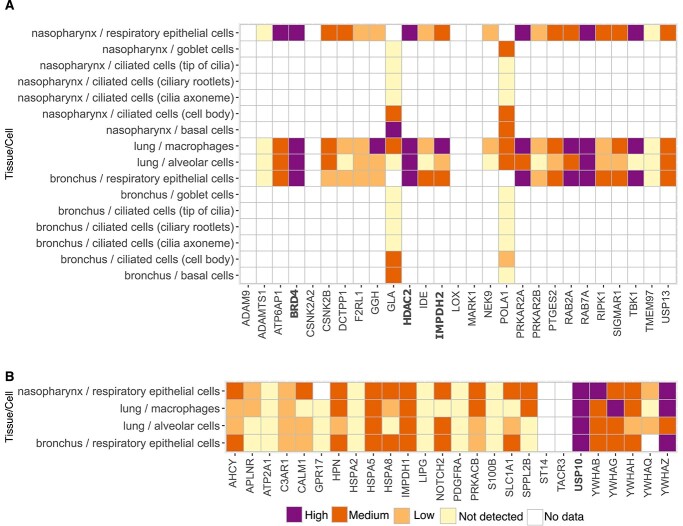
Protein expression in cells of upper and lower respiratory tract across the (**A**) 27 VIP targets and (**B**) 25 NIP targets. The expression classes originate from the Human Protein Atlas (colour legend).

Among the VIP targets, we found that BRD4 [bromodomain and extraterminal (BET) protein 4], RAB7A (Ras-related protein 7A), HDAC2 (Histone deacetylase 2) and IMPDH2 (Inosine-5-monophosphate dehydrogenase 2) were relatively highly expressed in the respiratory epithelial cells of the nasopharynx and bronchus, as well as in the lung macrophages and alveolar cells (Figure 4A). We de-prioritized RAB7A, because of its key role in the maturation of late endosomes, which are not among the primary sites through which SARS-CoV-2 enters human lung cells; most SARS-CoV-2s enter lung cells through fusion at the plasma membrane [28], while for the Omicron variants of concern (VOC), the virus may also use the endosomal route [29, 30].

Among the NIP targets, USP10 (Ubiquitin-specific peptidase 10) and YWHAZ (Tyrosine 3-Monooxygenase/Tryptophan 5-Monooxygenase Activation Protein Zeta) showed high-to-medium expression in respiratory epithelial cells of the nasopharynx and bronchus and in macrophages and alveolar cells of lung tissue (Figure 4B). Even though only preclinical inhibitors of these NIP host protein targets are currently available, we nonetheless pursued USP10 in the experimental validation, as this protein plays a role in stress granules and RNA processing, storage and degradation that are used by flaviviruses such as West Nile and dengue virus [31].

Based on the expression analyses of the protein targets in the VIP and NIP sets, we shortlisted BRD4 that is targeted by the SARS-CoV-2 envelope protein E, along with HDAC2 and IMPDH2 that are targeted by the viral protease (NSP-5) and exoribonuclease (NSP-14), respectively (Table 1). Among the NIP targets, we focused on USP10 in the experimental validation, since it engages the host VIP proteins G3BP1 and G3BP2 that directly engage the viral N protein [14].

**Table 1:** List of VIPs and NIPs shortlisted for the experimental assessment of compound effects

Protein class	Host protein	Viral protein	Degree	Betweenness centrality	Implicated in other viruses	Associated role
Epigenetic regulators	BRD4	E	26	1.46 × 10^−3^	HPV-1/2/5/7/9/10, EEPV4, HHV8, CRPV2	Regulate genes crucial for cell cycle, progression, inflammation and immune response
	HDAC2	Nsp5	27	7.99 × 10^−4^	Adeno-associated dependoparvovirus A, HPV, FLUAV	Plays an important role in transcriptional regulation, cell cycle progression and developmental events
Enzymes	IMPDH2	Nsp14	7	1.07 × 10^−4^	Dengue virus, HSV-1, HIV-1, FLUAV, Measles morbillivirus	Plays an important role in the regulation of cell growth and purine metabolic processes
	USP10^*^	N	8	1.75 × 10^−4^	HHV 8, FLUAV, Zika virus	Plays a role in regulation of autophagy

^*^This NIP interacts with VIP G3BP1/G3BP2.

### Experimental assessment of the host target inhibition on virus infection

Our computational prioritization approach identifies host targets that are expected to modulate virus infection within the SARS-CoV-2–host PPI network; however, similar to other network-based models, it does not predict whether the inhibition of the host targets leads to either virus suppressive or enhancing effects, nor whether the host target inhibition results in any toxic side effects. Therefore, in the experimental validation, we tested not only the effects of the host-targeting compounds on SARS-CoV-2 infection but also their effects on the viability of non-infected control cells.

We experimentally tested seven selected compounds that inhibit the VIPs BRD4, IMPDH2 and HDAC2, along with spautin-1, which inhibits the NIP USP10 and the VIP USP13 (Table 2). The compounds were first tested for modulation of SARS-CoV-2 infection in HEK293T cells that overexpress the key SARS-CoV-2 host factors ACE2 and TMPRSS2 [32]. Confirmatory experiments were performed in Calu 3 human lung cells [33], which are well-established targets for SARS-CoV-2 [28].

### Discovery of host modulators that enhance SARS-CoV-2 infection

**Figure 5:**
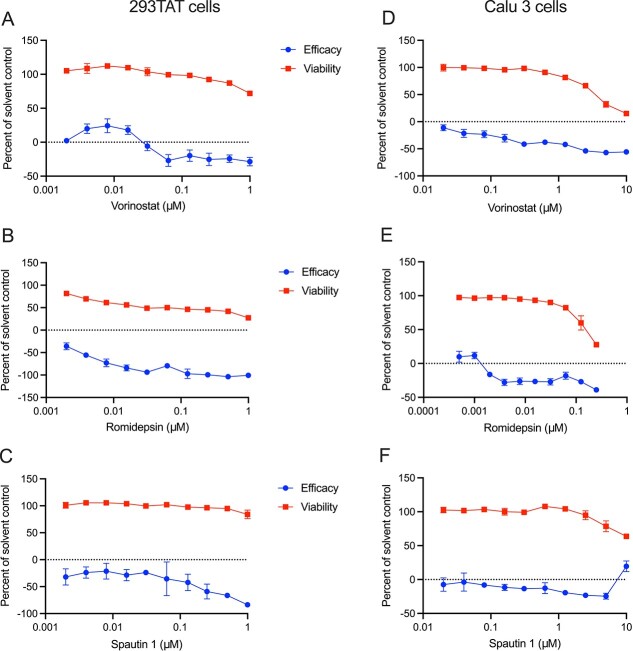
(**A**–**C**) Compounds that inhibit HDACs and USP10/13 enhance CPE during virus infection in 293TAT cells (i.e. appear to have proviral effects). Cells were treated with the indicated concentrations of compounds for 2 h prior to infection with SARS-CoV-2 at a MOI of 0.01. Parallel wells contained cells treated only with compounds to study toxicity. Forty-eight hours post infection, cell viability was assessed using the CellTiter-Glo assay and effects on virus-infected cells (efficacy) and non-infected cells (viability) were calculated as described in Materials and Methods. Data points reflect average and standard deviations of triplicate experiments per condition. (D-F) Confirmatory assays in Calu 3 cells. Cells were treated with the indicated concentrations of compounds for 2 h prior to infection with SARS-CoV-2 at a MOI of 0.1. Parallel wells contained cells treated only with compounds. Ninety-six hours post infection, cell viability was assessed using the CellTiter-Glo and effects on virus infection (efficacy) and viability were calculated as described in Materials and Methods. Data points reflect average and standard deviations of triplicate experiments per condition.

We noticed that several compounds that target chromatin regulating proteins, such as BRD4 and HDAC2 and deubiquitinating enzymes USP10 and USP13, did not show significant antiviral activity in our disease system (Figure 5, efficacy curve). In contrast, inhibition of these proteins actually increased virus-induced cytopathic effect (CPE) in the virus-infected cells, when compared to the DMSO control. This increased CPE was reflected in a negative antiviral efficacy (see Materials and Methods), and suggest that these compounds enhanced virus infection. This enhanced CPE (proviral effect) occurred at concentrations that were not directly cytopathic to non-infected cells (Figure 5, viability curve).

**Table 2:** Compounds tested in 293T-ACE2-TMPRSS2 and Calu 3 cells in the present work

Clinical phase	Compound name	ChEMBL ID	Host targets	Mechanism of action	ATC^a^ classification	Target bioactivity (nM)^b^
Investigational	Spautin-1	CHEMBL2391504	**USP10**/USP13	USP10/13 deubiquitinating activity inhibitor		600/600
				Autophagy inhibitor		
	JQ1	CHEMBL1957266	**BRD4**	BET bromodomain inhibitor		79.39
In clinical trial (II)	Merimepodib	CHEMBL304087	**IMPDH2**	Inosine-5′-monophosphate dehydrogenase 2 inhibitor		10.33
Approved	Mycophenolic acid	CHEMBL866	IMPDH1/**IMPDH2**	Inosine-5′-monophosphate dehydrogenase inhibitor	Immunosuppressant	37^*^/8.57^*^
	Vorinostat	CHEMBL98	**BRD4/HDAC2**	Histone deacetylase 1/2/3/6 inhibitor	Antineoplastic agent	375/15.69^*^
	Romidepsin	CHEMBL343448	**BRD4/HDAC2**	Histone deacetylase inhibitor	Antineoplastic agent	36/0.038^*^
	Fedratinib	CHEMBL1287853	**BRD4**/CSNK2A2/ NEK9 MARK1/TBK1	Tyrosine-protein kinase receptor FLT3 inhibitor	Antineoplastic agent	164^**^/120^**^/150^**^/700^**^/92^**^
				Tyrosine-protein kinase JAK2 inhibitor		

The boldfaced host targets were identified by the network approach.

^a^Anatomical Therapeutic Chemical; ^b^IC50/EC50 or ^*^K_i_, ^**^K_d_ from cell-based and biochemical assays in the ChEMBL database (31).

Compounds that showed such proviral phenotype included vorinostat, a broad range inhibitor of histone deacetylases (HDACs) (Figure 5A), and romidepsin, a more selective HDAC inhibitor that targets HDAC1 and HDAC2 (Figure 5B). Similarly, the investigational compound, spautin-1, which inhibits the NIP USP10 and VIP USP13, enhanced CPE at concentrations that were not inherently toxic to non-infected cells (Figure 5C). To further investigate this putative proviral effect, we tested these compounds during SARS-CoV-2 infection also in Calu 3 human lung cells, where the compounds again enhanced CPE during virus infection at concentrations that were not toxic to non-infected Calu 3 cells (Figure 5D–F).

**Figure 6:**
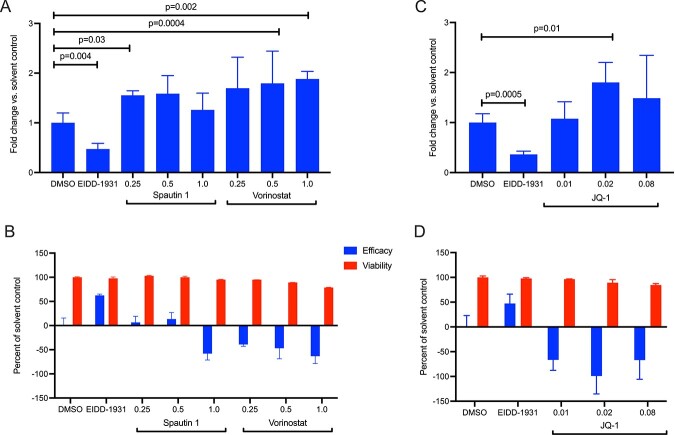
Spautin 1, vorinostat and JQ1 enhance SARS-CoV-2 infection. 293TAT cells were treated with the indicated concentrations of compounds prior to infection with SARS-CoV-2 at a MOI of 0.01. Parallel wells contained cells treated only with compounds. Twenty-four hours post infection, RNA was extracted from one plate and the relative abundance of viral RNA was determined by qRT-PCR (**A** and **C**). Forty-eight hours post infection, cell viability was assessed using the CellTiter-Glo assay and effect on virus-infected cells (efficacy) and non-infected cells (viability) were calculated (**B** and **D**). Data points in **A** and **B** reflect average and standard deviations of triplicate and quadruplicate experiments per condition, respectively. Statistical testing was done with a Student’s *t*-test (two-tailed).

If these compounds enhance virus infection, one would expect that they similarly enhance viral RNA expression in the infected cells. We therefore measured the level of SARS-CoV-2 RNA expression by qRT-PCR at 24 h post-infection of 293TAT cells, and parallel plates were assayed for CPE at 48 h as per our protocol. The qRT-PCR assays showed that both spautin-1 and vorinostat conferred an apparent proviral effect as they increased the abundance of viral RNA (Figure 6A), consistent with their enhancement of infection seen in the CPE assay (Figure 6B). These data confirm that spautin-1 and vorinostat enhance virus infection, and have proviral effects, and suggest that their host targets may therefore have antiviral function.

To further study the proviral effects of epigenetic regulators, we next examined the BRD4 inhibitor JQ1 in our CPE and viral RNA assays during infection of 293TAT cells. Similar to the other chromatin modifying enzyme inhibitors, JQ1 increased SARS-CoV-2 RNA expression and conferred a proviral effect (Figure 6C and D). Collectively, these observations suggest that alterations in chromatin regulation may regulate susceptibility for SARS-CoV-2. Interestingly, fedratinib, a selective oral JAK2 inhibitor recently approved in the United States for treatment of adult patients with myelofibrosis [34], which has off-target activity against the VIPs BRD4, TBK1, MARK1 and CSNK2A2, also displayed a similar proviral effect in our disease system (Supplementary Figure S2).

### Network approach identifies context-specific inhibitors of virus infection

Since a compound’s response is often highly context-dependent, it is important to consider its effects also in other cell lines and assays when determining the influence of a host target modulation on viral infection. Therefore, we cross-compared the host-targeting compounds identified by our network-based analysis with the profiling studies carried out by the National Centre for Advancing Translational Sciences (NCATS) [35] with live virus infectivity CPE assay. The NCATS has tested a wide collection of 9187 compounds consisting of several approved, anti-infective compounds that have been reported in literature and with target information. To date, 7.7% (730/9187) of the compounds have been classified as antivirals based on the NCATS criteria in Vero E6 cell lines (compounds with curve rank >0).

A total of 260 compounds from our network-based analyses were included in the NCATS compound list, and based on the NCATS CPE assay, 18.1% (47/260) of the identified compounds were classified as antivirals (Supplementary Table S4 available online at http://bib.oxfordjournals.org/). Therefore, the network approach led to a 2.4-fold improvement in success rate, compared to the 7.7% success rate of the NCATS compound screen. As a comparison, we also benchmarked the predictions from another computational method that integrates three network-based drug repurposing algorithms [13]. Among the 73 overlapping compounds, 21.9% (16/73) were validated as antivirals by the NCATS assay (see Supplementary Text for details available online at http://bib.oxfordjournals.org/). The accuracies of these two computational methods provide a reasonable upper bound of what can be expected when using network-based protein prioritization methods.

We further cross-referenced the compounds identified by our method with the antivirals reported through various publications using various assays and cell lines and catalogued in the Coronavirus resistance database CoV-RDB database (Supplementary Table S4 available online at http://bib.oxfordjournals.org/) [36]. For instance, imatinib, an approved inhibitor of Bcr-Abl kinase, which has off-target activity against the NIP PDGFRA, showed antiviral efficacy in VeroE6 cells and human airway epithelial cultures (Table 3) [37, 38]. Imatinib was advanced to clinical trials, albeit without evidence for clinical efficacy (ClinicalTrials.gov, trial numbers NCT04794088, NCT04394416) [39].

**Table 3:** Compounds identified by our network-based approach and validated as antivirals in other studies

Compound	Host targets	Target class	Cell line	Efficacy (%)	SI	EC50 (μM)	Resource
Mycophenolic acid	IMPDH1	VIP	VeroE6	45.67	>147	0.9	1, 2
Merimepodib	IMPDH1/2		VeroE6	72.28			1
Fedratinib	BRD4/CSNK2A2/NEK9 MARK1/TBK1		Huh 7.5		83	0.02	2
Romidepsin	BRD4/HDAC2		HEK293T		194.4	0.09 ± 0.05	3
Linifanib	PDGFRA	NIP	VeroE6	30.84			1
Bemcentinib			VeroE6/Calu-3 Huh7.5	73.13	7.8/47	2.1/0.1	1, 2
Imatinib			VeroE6/HAE	92.09/100.7117.51	16, >9.5, >5.8	2.5, 3.2 5.3/>10	1, 2
Pazopanib			VeroE6	43.66			1
Masitinib			VeroE6	63.83	12	2.3	1, 2
Sorafenib			VeroE6	43.12			1
OSI-632			VeroE6	42.27			1
Nafamostat	HPN/ST14		Caco2/VeroE6/Calu-3		>500/>4.4	0.04/23/0.01	2
Quizartinib	PRKACB		VeroE6	43.88			1
SB-202190			VeroE6	117.73			1
Enzastaurin			VeroE6	63.93			1

Supplementary Table S4 lists all the compounds identified in NCATS resource.

SI, the compound concentration toxic to cells (CC50/EC50). EC50, the compound concentration required to inhibit virus replication by 50%. CC50, the compound concentration required to reduce the cell viability by 50% compared to uninfected control from NCATS or CoV-RDB resource.

Resource: 1, NCATS; 2, Stanford University Coronavirus Antiviral & Resistance Database (CoV-RBD); 3, Liu *et al*. (2020).

Another example of effective antiviral that targets TMPRSS2 and the NIPs HPN and ST14 is nafamostat, an anticoagulant drug that was shown to block SARS-CoV-2 infection in Calu-3, Caco2 and VeroE6 cells (Table 3). This compound was also advanced to clinical trials in the RACONA study (NCT04352400) to test its efficacy in lowering lung function deterioration and reducing intensive care admissions in COVID-19 patients. However, intravenous administration of nafamostat mesylate in a randomized controlled trial did not show evidence as an effective treatment for COVID-19 in a limited cohort of 42 patients, but it was anyway suggested for further investigation as an early treatment option for COVID-19 [40].

The IMPDH inhibitors mycophenolic acid (MPA) and merimepodib have previously been shown to inhibit SARS-CoV-2 infection in VeroE6 cells [41–43]. MPA also showed antiviral effect in lung organoids and in nasal and bronchial epithelial explants (Table 3) [44, 45]. In our CPE assay, MPA and merimepodib showed a modest but reproducible antiviral effect, and no apparent toxicity in 293TAT cells (Supplementary Figure S3 available online at http://bib.oxfordjournals.org/). These results indicate differences in antiviral effects between cell lines, experimental assays and studies, which need to be considered when interpreting hits from any compound or target prediction method. Such cell context specificity complicates the reuse of published data of antiviral effects for the validation of cell line-specific computational predictions, making own experimental assays critical.

## Discussion

Over the past 2 years, hundreds of predictive tools for COVID-19 diagnosis and prognosis have been developed based on the accumulated proteomic, transcriptomic and clinical data sets. However, based on systematic literature reviews, most of the predictive approaches developed to support medical decision-making suffer from training data of low quality and poor reporting of the models [46]. For instance, so far none of the models for prediction of clinical outcome developed using chest radiographs and CT scans are of potential clinical use, mainly due to methodological flaws and high or unclear risk of bias in the imaging datasets used for the model training [47]. Compared to supervised prediction models, network-based models provide a holistic view of the biological system being studied (here, virus–host interactions), and do not require large amount of outcome data for model training. Network-based approaches can also broaden our understanding of the mechanisms of viral infections, compared to black-box machine learning models.

Consistent with previous studies, we demonstrated that VIPs tend to be in essential positions for PPI network information flow. Gysi *et al*. demonstrated that most of the drugs that successfully reduced viral infection do not bind the proteins targeted by SARS-CoV-2, indicating that these drugs rely on network- or pathway-level mechanisms that cannot be identified using docking-based strategies [13]. We therefore expanded the potential target space to include additional host proteins that can be useful for disease pathogenesis but may not be directly targeted by viral proteins. Along with cell context specificity, it was found important to consider interactions of the host proteins, as they can play pivotal roles in virus modulation. To elucidate the importance of the host proteins identified by the network approach, we screened for their effects on SARS-CoV-2 infection using selective inhibitors of the cellular proteins.

**Figure 7:**
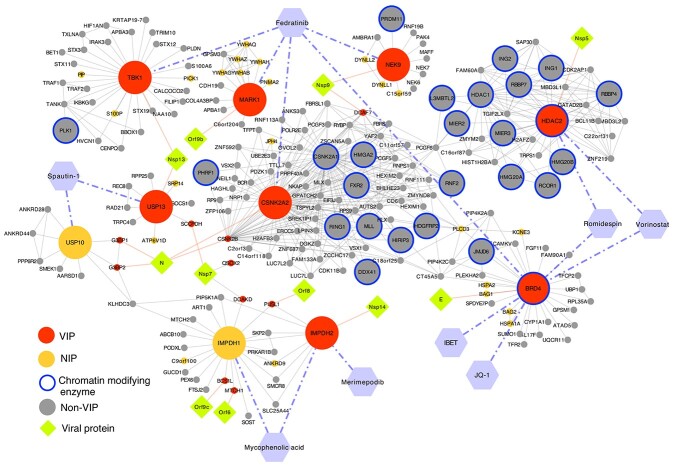
Network representation of selected targets and their protein–protein and compound–protein interactions with the validated compounds. Targets and their nearest neighbours are shown in the network.

Interestingly, our study identified several antiviral proteins by virtue of the compounds inhibiting their targets acting as proviral agents. In particular, inhibition of epigenetic regulators BRD4 and HDAC2 by JQ1, vorinostat and romidepsin resulted in an increase in viral RNA, suggesting that these proteins function in an antiviral fashion (Figure 6). The viral protein Nsp 5, which targets HDAC2 (Table 1), cleaves the SARS-CoV-2 polyprotein and has developed strategies to inhibit the transport of HDAC into the nucleus and potentially affect the ability of HDAC2 to mediate inflammation and interferon response [14]. Also, HDAC2 has been highlighted as one of the potential determinants of age-related susceptibility to SARS-CoV-2 infection, since it is downregulated in lungs of older individuals [48]. HDAC2 and BRD4 interact with several other epigenetic modifiers in the SARS-CoV-2 network (Figure 7). HDAC2 erases acetyl marks on histones H3 and H4 to promote predominantly repression of transcription, and BRD4 is a histone acetyl reader that is predominantly an activator of transcription. Epigenetic modifications are significant in regulating cellular function, both in health and in disease. Chen *et al*. showed that the viral protein E in its acetylated form can directly bind to the second bromodomain of BRD4, and it has evolved to antagonize interferon responses via inhibition of BET proteins [49].

The epigenetic modulators play a diverse role in viral infection and viruses have developed strategies to evade pathways involving these proteins for their gain [50, 51]. For instance, HDAC2 and BRD4 regulate a multitude of genes, including the antiviral response genes, and their inhibition is generally growth-suppressive, which may explain the proviral effect of their inhibition in the context of the SARS-CoV2 infection. Another important factor to consider when investigating the enhancing and suppressive effects of targets and compounds on SARS-CoV-2 infection is the cell context specificity of protein interactions and the dependency of the compound effect on the cell context. Additional studies will be required to define how HDAC2 and BRD4, and the multitude of downstream genes that they regulate, modulate SARS-CoV-2 infection.

Recently, Daniloski *et al*. showed a proviral effect of vorinostat in human alveolar basal epithelial carcinoma cells expressing ACE2 [52]. On the other hand, computational analysis using network clustering along with connectivity map (C-Map) analysis of SARS-COV-2 PPI network identified vorinostat as potentially antiviral [53]. Such contradicting results may be due to dependency of the compound’s effect on the cell context. To explore cell context specificity, we tested JQ1 and IBET151, two inhibitors of BRD4, in Calu-3 cells and observed that the compounds showed antiviral effects, but with more pronounced toxicity in non-infected cells (Supplementary Figure S4 available online at http://bib.oxfordjournals.org/), making their clinical application challenging.

The deubiquitinase proteins USP10 and USP13 may act as antiviral cellular proteins, since spautin-1, an autophagy inhibitor that targets these ubiquitinases, enhances SARS-CoV-2 infection. *In vivo* and *in vitro* studies have shown that SARS-CoV-2 inhibits autophagy in infected cells, suggesting the use of compounds that induce autophagy as an antiviral treatment strategy [54]. The roles of USP10 and USP13 in SARS-CoV-2 infection therefore warrant further investigation. RAB7A is another protein that might be worth pursuing for some variants of concern (VOCs), such as Omicron that also use the endosomal entry pathway [29].

Inhibiting a host target alone is unlikely to have the potency one can achieve by inhibiting a viral protein such as a viral RNA polymerase. However, host-targeted antiviral (HTA) agents exploit the dependence of the virus on specific host proteins and pathways during replication, and hence drug resistance may be less likely with the HTAs. Combination therapies offer widespread and well-documented advantages in the treatment of complex diseases such as cancer, HIV and HCV [55–57], and combining DAA and HTA agents may offer an effective way to combat drug resistance. Since we observed a weak antiviral activity for the IMPDH inhibitors MPA and merimepodib (Supplementary Figure S3 available online at http://bib.oxfordjournals.org/), we further explored whether their activity could be improved by combination with molnupiravir (MPV), a directly acting antiviral (DAA) that targets the viral RNA-dependent RNA polymerase, which has been approved for emergency use by the US Food and drug Administration [58, 59]. We evaluated the two-drug combinations against SARS-CoV-2 infections in 293TAT cells. The combinations led to a minimal synergistic effect, but importantly, there was no detectable toxicity in non-infected cells in either of the combination assays (Supplementary Figure S5 available online at http://bib.oxfordjournals.org/).

The need for effective antiviral drugs is increasing but only a few viral enzymes such as polymerases, polymerase co-factors and proteases are available for targeting with DAAs, and these are used as DAA combination therapies for HIV and HCV [60, 61]. Despite their increasing threat of causing infectious diseases, we still do not have many widely available, effective antivirals for SARS-CoV-2, SARS-CoV-1, Dengue, Ebola, Lassa and others [62]. Moreover, the interconnected nature of our societies, coupled with environmental disruption and habitat encroachment, magnifies these threats. While employing HTAs is potentially more challenging, novel approaches such as synthetic lethality may offer effective and safe solutions for more selective treatment options by inhibiting virus-infected cells without affecting non-infected cells, as has been earlier shown in cancer cells [63], and recently for viruses [64–66]. Thus, we argue that HTA agents should remain as part of the development pipeline. We especially posit that combining HTAs with effective DAAs, such as molnupiravir and paxlovid, can provide an effective antiviral strategy to combat SARS-CoV-2 infection.

In summary, we have demonstrated that a network-based approach of SARS-CoV-2 and VIPs and NIPs can be leveraged to prioritize host modulators of viral infection. However, both the compound sensitivity assays and PPIs can be highly context-specific, hence it is important to consider the molecular characteristics in relevant cell lines or *in vivo* models to provide more actionable findings. Furthermore, testing of the compounds in non-infected cells is critical for avoiding broadly toxic compound responses and to identify either safer small molecules for therapeutic applications or selective chemical tools to probe virus–host interactions that regulate virus infection. The network model predictions could be further improved by leveraging the information from compound–target binding interactions already in the network modelling phase, along with the use of gene expression datasets across different cell lines perturbed by the explored drugs, using the C-MAP approach.

## Materials and methods

### SARS-CoV-2 PPI network construction

The SARS-CoV-2 PPIs were extracted from Gordon *et al.* [14]. The PPIs include physical interaction among 26 viral proteins and 332 human proteins identified using affinity purification mass spectrometry in HEK293T cell line. We constructed the SARS-CoV-2 network by overlaying the HEK293T PPI network with the interactions from the BioPlex Interactome [15]. Out of the 332 viral-targeted host proteins, called viral interacting proteins (VIPs), 298 had an interaction in the BioPlex database. For each of the 298 VIPs, we considered the protein interaction partners that were directly interacting with the viral-targeted proteins, that is, their nearest neighbours, thus creating an induced subgraph from the BioPlex HEK293T network. The final SARS-CoV-2 interaction network included 3978 proteins and 41 015 interactions, which were used for further downstream analysis. This PPI network is context-specific, as we incorporated PPIs from HEK293T cell line that was used by Gordan *et al.* [14], along with additional proteins that are closest to the VIPs in terms of their connectivity to VIP proteins.

### Identification of host targets using protein prioritization

We used a network-based protein prioritization approach, the RWR, to identify host protein targets when starting from the VIP source protein set. We used Arete Cytoscape plugin that provides an exact closed-form solution, rather than an approximate or iterative solution for RWR, which is robust to the restart probability parameter (*r* was set here equal to 0.7, which is the recommended default value) [19, 67] (See Supplementary Text for further details available online at http://bib.oxfordjournals.org/).

### Biological characterization of the network proteins

The GO and KEGG pathway enrichment analysis for the VIP and NIP sets were carried out using the clusterProfiler R package [68]. The protein names were mapped to the NCBI entrez ID using the genome-wide annotation for human (org.Hs.eg.db) R package [69]. The tissue expression of the proteins in the network were analysed using HPAanalyze [70], an R package that provides functions for retrieving, exploratory analysing and visualizing the Human Protein Atlas data [27].

### Identification of potent compounds for the host targets

The predefined host target protein sets (VIPs and predicted NIPs) were queried in the ChEMBL database [26], a public bioactivity data repository, to find compounds that inhibit the identified host targets. A compound is active against a protein target when it binds to it and inhibits a specific molecular pathway. The strength of this inhibition can be quantified using several affinity measurements, such as IC_50_ and EC_50_, that refer to the *in vitro* concentration of the compound required to inhibit half of the target volume or produce half of the overall effect, respectively. The binding affinity that a compound has against a protein target can be defined by *K*_i_ (the inhibition constant) or *K*_d_ (the dissociation constant). We considered compounds with a bioactivity of less than 1000 nM against the VIP or NIP targets as potent compounds for these host targets. In cases where multiple compounds inhibit a particular host protein, we selected the compounds with the highest potency (lowest bioactivity value) as inhibitors for experimental validations.

### Experimental validation

Infectious SARS-CoV-2 was obtained from BEI Resources (Isolate USA-WA1/2020 NR-52281). 293TAT cells were plated the day prior to infection, while Calu3 cells were plated 2 days prior to infection. Compounds at various dose ranges were then added to plates and infectious SARS-CoV-2 was added to appropriate wells. Multiplicity of infection (MOI) of live SARS-CoV-2 is indicated in each figure legend. Mock-infected control wells received standard medium. Plates were then incubated at 37°C, 5% CO_2_ for 48 (293TAT) or 96 (Calu 3) h. CellTiter-Glo (CTG) assay (Promega, G9243) was used to measure cell viability in virus-infected and drug-treated wells and in parallel mock-infected and drug-treated wells. The assay measures the number of viable cells in culture by quantifying ATP, indicating the presence of metabolically active cells. Luminescence was read on a Biotek Synergy H4 plate reader. Each condition was conducted in triplicate. For further experimental details, please see Supplementary Text available online at http://bib.oxfordjournals.org/.

**Key Points**

- Viruses exploit host machinery, making it important to understand the virus–host dependencies to gain better insight of the key regulators of viral infection.
- Using a context-specific SARS-COV-2–host PPI network, a computational framework was developed to identify host modulators of viral infection.
- Chromatin modifying host proteins HDAC2 and BRD4, along with deubiquitinating enzyme USP10, acted as antiviral proteins.
- IMPDH inhibitors mycophenolic acid and merimepodib showed modest antiviral response to SARS-COV-2 infection, and no toxic effects.
- Cell context specificity is a critical factor when identifying selective modulators of viral infection and potential antiviral therapeutics.
- Topology-based network models cannot distinguish between host proteins, the inhibition of which leads to either virus suppressive or enhancing effects.

## Supplementary Material

SUPPLEMENTARY_FIGURES_bbac456

Supplementary_Table_S1_bbac456

Supplementary_Table_S2_bbac456

Supplementary_Table_S3_bbac456

Supplementary_Table_S4_bbac456

SUPPLEMENTARY_TEXT_bbac456

## Data Availability

The interactions for the network construction are provided in Supplementary File 1. The codes and data for running the network construction algorithm are available on github: https://github.com/ocbe-uio/discovering-host-viral-modulators

## Author Biographies

**Vandana Ravindran** is a postdoctoral researcher at Oslo Centre for Biostatistics and Epidemiology (OCBE), University of Oslo and at the Institute for Cancer Research, Oslo University Hospital. Her work spans computational biology, bioinformatics, complex networks and omics data integration.

**Jessica Wagoner** is a research scientist in the Department of Laboratory Medicine & Pathology, University of Washington. Her research focuses on virology.

**Paschalis Athanasiadis** is a PhD candidate at OCBE, University of Oslo and at the Institute for Cancer Research, Oslo University Hospital. His research areas are computational drug screening and bioinformatics.

**Andreas B. Den Hartigh** is a research scientist in the Department of Laboratory Medicine & Pathology, University of Washington. His research focuses on the regulation of caspase-1 activation following infection by pathogens.

**Julia M. Sidorova** is an associate professor in the Department of Laboratory Medicine & Pathology, University of Washington. Her research areas are DNA replication and epigenetics.

**Aleksandr Ianevski** is a PhD candidate at FIMM, HiLIFE, University of Helsinki. His research areas are combinatorial drug screening and prediction of selective drug combinations.

**Susan L. Fink** is an associate professor at the Department of Laboratory Medicine & Pathology, University of Washington. Her research interests are clinical immunology, cellular and molecular virology and innate immunity.

**Arnoldo Frigessi** is a professor at OCBE, University of Oslo. His work focuses on biostatistics, stochastic models and graph theory.

**Judith M. White** is a professor emeritus at the Department of Cell Biology and Department of Microbiology, University of Virginia. Her research interests are in virus entry and drug combinations for pandemic preparedness.

**Stephen J. Polyak** is a professor at the Department of Laboratory Medicine & Pathology, University of Washington. His research focuses on the interactions between cells and viruses, cellular innate antiviral and inflammatory responses to virus infection, and drug combinations for pandemic preparedness.

**Tero Aittokallio** is a professor at OCBE, University of Oslo, and group leader at the Institute for Cancer Research and FIMM, HiLIFE unit of University of Helsinki. His research focuses on network pharmacology and statistical modelling of multi-omics data.

## References

[1] Mattoo S-S , Kim S-J, Ahn D-G, et al. Escape and over-activation of innate immune responses by SARS-CoV-2: two faces of a coin. Viruses 2022;14(3):530. 35336937 10.3390/v14030530 PMC8951629

[2] Shoemaker JE , Fukuyama S, Eisfeld AJ, et al. Integrated network analysis reveals a novel role for the cell cycle in 2009 pandemic influenza virus-induced inflammation in macaque lungs. BMC Syst Biol 2012;6:1–14. 22937776 10.1186/1752-0509-6-117 PMC3481363

[3] Heaton NS , Moshkina N, Fenouil R, et al. Targeting viral proteostasis limits influenza virus, HIV, and dengue virus infection. Immunity 2016;44:46–58. 26789921 10.1016/j.immuni.2015.12.017 PMC4878455

[4] Yang SL , DeFalco L, Anderson DE, et al. Comprehensive mapping of SARS-CoV-2 interactions in vivo reveals functional virus-host interactions. Nat Commun 2021;12:1–15. 34433821 10.1038/s41467-021-25357-1 PMC8387478

[5] O’Donoghue SI , Schafferhans A, Sikta N, et al. SARS-CoV-2 structural coverage map reveals viral protein assembly, mimicry, and hijacking mechanisms. Mol Syst Biol 2021;17:e10079. 34519429 10.15252/msb.202010079 PMC8438690

[6] Du H , Chen F, Liu H, et al. Network-based virus-host interaction prediction with application to SARS-CoV-2. Patterns 2021;2:100242. 33817672 10.1016/j.patter.2021.100242 PMC8006187

[7] Chen Z , Wang C, Feng X, et al. Interactomes of SARS-CoV-2 and human coronaviruses reveal host factors potentially affecting pathogenesis. EMBO J 2021;40:e107776. 34232536 10.15252/embj.2021107776 PMC8447597

[8] Karunakaran KB , Balakrishnan N, Ganapathiraju MK. Interactome of SARS-CoV-2 modulated host proteins with computationally predicted PPIs: insights from translational systems biology studies. Front Syst Biol 2022;2:815237.

[9] Verstraete N , Jurman G, Bertagnolli G, et al. CovMulNet19, integrating proteins, diseases, drugs, and symptoms: a network medicine approach to COVID-19. Network and systems medicine 2020;3:130–41. 33274348 10.1089/nsm.2020.0011 PMC7703682

[10] Sadegh S , Matschinske J, Blumenthal DB, et al. Exploring the SARS-CoV-2 virus-host-drug interactome for drug repurposing. Nat Commun 2020;11:1–9. 32665542 10.1038/s41467-020-17189-2 PMC7360763

[11] Ostaszewski M , Niarakis A, Mazein A, et al. COVID19 Disease Map, a computational knowledge repository of virus–host interaction mechanisms. Mol Syst Biol 2021;17:e10387. 34664389 10.15252/msb.202110387 PMC8524328

[12] Muratov EN , Amaro R, Andrade CH, et al. A critical overview of computational approaches employed for COVID-19 drug discovery. Chem Soc Rev 2021;50:9121–51. 34212944 10.1039/d0cs01065k PMC8371861

[13] Morselli Gysi D , do Valle Í, Zitnik M, et al. Network medicine framework for identifying drug-repurposing opportunities for COVID-19. Proc Natl Acad Sci 2021;118:e2025581118. 33906951 10.1073/pnas.2025581118 PMC8126852

[14] Gordon DE , Jang GM, Bouhaddou M, et al. A SARS-CoV-2 protein interaction map reveals targets for drug repurposing. Nature 2020;583:459–68. 32353859 10.1038/s41586-020-2286-9 PMC7431030

[15] Huttlin EL , Bruckner RJ, Navarrete-Perea J, et al. Dual proteome-scale networks reveal cell-specific remodeling of the human interactome. Cell 2021;184:3022–40. 33961781 10.1016/j.cell.2021.04.011 PMC8165030

[16] Dyer MD , Murali T, Sobral BW. The landscape of human proteins interacting with viruses and other pathogens. PLoS Pathog 2008;4:e32. 18282095 10.1371/journal.ppat.0040032 PMC2242834

[17] Brito AF , Pinney JW. Protein–protein interactions in virus–host systems. Front Microbiol 2017;8:1557. 28861068 10.3389/fmicb.2017.01557 PMC5562681

[18] Yuryev A . Pathway analysis in drug discovery. In: Systems Biology in Drug Discovery and Development, 2011, 287–302. John Wiley & Sons, Inc., Hoboken, New Jersey.

[19] Lysenko A , Boroevich KA, Tsunoda T. Arete–candidate gene prioritization using biological network topology with additional evidence types. BioData mining 2017;10:1–12. 28694847 10.1186/s13040-017-0141-9 PMC5501438

[20] Grove J , Marsh M. The cell biology of receptor-mediated virus entry. J Cell Biol 2011;195:1071–82. 22123832 10.1083/jcb.201108131 PMC3246895

[21] Walsh D , Mohr I. Viral subversion of the host protein synthesis machinery. Nat Rev Microbiol 2011;9:860–75. 22002165 10.1038/nrmicro2655 PMC7097311

[22] Romero-Brey I , Bartenschlager R. Endoplasmic reticulum: the favorite intracellular niche for viral replication and assembly. Viruses 2016;8:160. 10.3390/v8060160 PMC4926180 27338443

[23] V’kovski P , Gerber M, Kelly J, et al. Determination of host proteins composing the microenvironment of coronavirus replicase complexes by proximity-labeling. elife 2019;8:e42037. 30632963 10.7554/eLife.42037 PMC6372286

[24] Dove B , Brooks G, Bicknell K, et al. Cell cycle perturbations induced by infection with the coronavirus infectious bronchitis virus and their effect on virus replication. J Virol 2006;80:4147–56. 16571830 10.1128/JVI.80.8.4147-4156.2006 PMC1440480

[25] Hassan Z , Kumar ND, Reggiori F, et al. How viruses hijack and modify the secretory transport pathway. Cell 2021;10:2535. 10.3390/cells10102535 PMC8534161 34685515

[26] Gaulton A , Hersey A, Nowotka M, et al. The ChEMBL database in 2017. Nucleic Acids Res 2017;45:D945–54. 27899562 10.1093/nar/gkw1074 PMC5210557

[27] Uhlén M , Fagerberg L, Hallström BM, et al. Tissue-based map of the human proteome. Science 2015;347:1260419. 25613900 10.1126/science.1260419

[28] Hoffmann M , Kleine-Weber H, Schroeder S, et al. SARS-CoV-2 cell entry depends on ACE2 and TMPRSS2 and is blocked by a clinically proven protease inhibitor. Cell 2020;181:271–80. 32142651 10.1016/j.cell.2020.02.052 PMC7102627

[29] Willett BJ , Grove J, MacLean OA, et al. SARS-CoV-2 Omicron is an immune escape variant with an altered cell entry pathway. Nat Microbiol 2022:7:1161–1179. https://doi.org/2022.01.03.21268111. 10.1038/s41564-022-01143-7 PMC9352574 35798890

[30] Peacock TP , Brown JC, Zhou J, et al. The SARS-CoV-2 variant, Omicron, shows rapid replication in human primary nasal epithelial cultures and efficiently uses the endosomal route of entry. bioRxiv 2022. https://doi.org/10.1101.2021.12.31.474653.

[31] Gaete-Argel A , Márquez CL, Barriga GP, et al. Strategies for success. Viral infections and membraneless organelles. Front Cell Infect Microbiol 2019;9:336. 10.3389/fcimb.2019.00336 PMC6797609 31681621

[32] Park Y-J , Acosta D, Vassell R, et al. D-dimer and CoV-2 spike-immune complexes contribute to the production of PGE2 and proinflammatory cytokines in monocytes. PLoS Pathog 2022;18:e1010468. 35385545 10.1371/journal.ppat.1010468 PMC9015149

[33] Biering SB , Van Dis E, Wehri E, et al. Screening a library of FDA-approved and bioactive compounds for antiviral activity against SARS-CoV-2. ACS Infect Dis 2021;7:2337–51. 34129317 10.1021/acsinfecdis.1c00017

[34] Talpaz M , Kiladjian J-J. Fedratinib, a newly approved treatment for patients with myeloproliferative neoplasm-associated myelofibrosis. Leukemia 2021;35:1–17. 32647323 10.1038/s41375-020-0954-2 PMC7787977

[35] Brimacombe KR , Zhao T, Eastman RT, et al. An OpenData portal to share COVID-19 drug repurposing data in real time. bioRxiv 2020. 10.1101/2020.06.04.135046.

[36] Tzou PL , Tao K, Pond SLK, et al. Coronavirus Resistance Database (CoV-RDB): SARS-CoV-2 susceptibility to monoclonal antibodies, convalescent plasma, and plasma from vaccinated persons. PLoS One 2022;17:e0261045. 35263335 10.1371/journal.pone.0261045 PMC8906623

[37] Aman J , Duijvelaar E, Botros L, et al. Imatinib in patients with severe COVID-19: a randomised, double-blind, placebo-controlled, clinical trial. Lancet Respir Med 2021;9:957–68. 34147142 10.1016/S2213-2600(21)00237-X PMC8232929

[38] Weston S , Coleman CM, Haupt R, et al. Broad anti-coronavirus activity of food and drug administration-approved drugs against SARS-CoV-2 in vitro and SARS-CoV in vivo. J Virol 2020;94:e01218–20. 32817221 10.1128/JVI.01218-20 PMC7565640

[39] Touret F , Driouich J-S, Cochin M, et al. Preclinical evaluation of imatinib does not support its use as an antiviral drug against SARS-CoV-2. Antivir Res 2021;193:105137. 34265358 10.1016/j.antiviral.2021.105137 PMC8274277

[40] Quinn TM , Gaughan EE, Bruce A, et al. Randomised controlled trial of intravenous nafamostat mesylate in COVID pneumonitis: phase 1b/2a experimental study to investigate safety, pharmacokinetics and pharmacodynamics. EBioMedicine 2022;76:103856. 35152152 10.1016/j.ebiom.2022.103856 PMC8831100

[41] Kato F , Matsuyama S, Kawase M, et al. Antiviral activities of mycophenolic acid and IMD-0354 against SARS-CoV-2. Microbiol Immunol 2020;64:635–9. 32579258 10.1111/1348-0421.12828 PMC7362101

[42] Bukreyeva N , Sattler RA, Mantlo EK, et al. The IMPDH inhibitor merimepodib provided in combination with the adenosine analogue remdesivir reduces SARS-CoV-2 replication to undetectable levels in vitro. F1000Research 2020;9:361.

[43] Schoot TS , Kerckhoffs APM, Hilbrands LB, et al. Immunosuppressive Drugs and COVID-19: A Review. Front Pharmacol 2020;11:1333. 32982743 10.3389/fphar.2020.01333 PMC7485413

[44] Murer L , Volle R, Andriasyan V, et al. Identification of broad anti-coronavirus chemical agents for repurposing against SARS-CoV-2 and variants of concern. Curr Res Virol Sci 2022;3:100019. 35072124 10.1016/j.crviro.2022.100019 PMC8760634

[45] Han Y , Yang L, Duan X, et al. Identification of candidate COVID-19 therapeutics using hPSC-derived lung organoids. bioRxiv 2020. 10.1101/2020.05.05.079095.

[46] Wynants L , Calster BV, Collins GS, et al. Prediction models for diagnosis and prognosis of covid-19: systematic review and critical appraisal. BMJ 2020;369:m1328. 32265220 10.1136/bmj.m1328 PMC7222643

[47] Roberts M , Driggs D, Thorpe M, et al. Common pitfalls and recommendations for using machine learning to detect and prognosticate for COVID-19 using chest radiographs and CT scans. Nature Mach Intell 2021;3:199–217.

[48] Chow RD , Majety M, Chen S. The aging transcriptome and cellular landscape of the human lung in relation to SARS-CoV-2. Nat Commun 2021;12:1–13. 33397975 10.1038/s41467-020-20323-9 PMC7782551

[49] Chen IP , Longbotham JE, McMahon S, et al. Viral E protein neutralizes BET protein-mediated post-entry antagonism of SARS-CoV-2. Cell Reports 2022;40:111088. 10.1016/j.celrep.2022.111088 PMC9234021 35839775

[50] Knipe DM , Lieberman PM, Jung JU, et al. Snapshots: chromatin control of viral infection. Virology 2013;435:141–56. 23217624 10.1016/j.virol.2012.09.023 PMC3531885

[51] Crimi E , Benincasa G, Figueroa-Marrero N, et al. Epigenetic susceptibility to severe respiratory viral infections and its therapeutic implications: a narrative review. Br J Anaesth 2020;125:1002–17. 32828489 10.1016/j.bja.2020.06.060 PMC7438995

[52] Daniloski Z , Jordan TX, Wessels H-H, et al. Identification of required host factors for SARS-CoV-2 infection in human cells. Cell 2021;184:92–105. 33147445 10.1016/j.cell.2020.10.030 PMC7584921

[53] Adhami M , Sadeghi B, Rezapour A, et al. Repurposing novel therapeutic candidate drugs for coronavirus disease-19 based on protein-protein interaction network analysis. BMC Biotechnol 2021;21:1–11. 33711981 10.1186/s12896-021-00680-z PMC7952507

[54] Gassen NC , Papies J, Bajaj T, et al. SARS-CoV-2-mediated dysregulation of metabolism and autophagy uncovers host-targeting antivirals. Nat Commun 2021;12:1–15. 34155207 10.1038/s41467-021-24007-w PMC8217552

[55] Sun W , Sanderson PE, Zheng W. Drug combination therapy increases successful drug repositioning. Drug Discov Today 2016;21:1189–95. 27240777 10.1016/j.drudis.2016.05.015 PMC4907866

[56] Shyr ZA , Cheng Y-S, Lo DC, et al. Drug combination therapy for emerging viral diseases. Drug Discov Today 2021;26:2367–76. 34023496 10.1016/j.drudis.2021.05.008 PMC8139175

[57] White JM , Schiffer JT, Bender Ignacio RA, et al. Drug combinations as a first line of defense against coronaviruses and other emerging viruses. MBio 2021;12:e03347–21. 10.1128/mbio.03347-21 PMC8689562 34933447

[58] Fischer WA , Eron JJ, Holman W, et al. A phase 2a clinical trial of molnupiravir in patients with COVID-19 shows accelerated SARS-CoV-2 RNA clearance and elimination of infectious virus. Sci Transl Med 2021;14:eabl7430. 10.1126/scitranslmed.abl7430 PMC10763622 34941423

[59] Wahl A , Gralinski LE, Johnson CE, et al. SARS-CoV-2 infection is effectively treated and prevented by EIDD-2801. Nature 2021;591:451–7. 33561864 10.1038/s41586-021-03312-w PMC7979515

[60] Cihlar T , Fordyce M. Current status and prospects of HIV treatment. Curr Opin Virol 2016;18:50–6. 27023283 10.1016/j.coviro.2016.03.004

[61] Sarrazin C . Treatment failure with DAA therapy: importance of resistance. J Hepatol 2021;74:1472–82. 33716089 10.1016/j.jhep.2021.03.004

[62] Meganck RM , Baric RS. Developing therapeutic approaches for twenty-first-century emerging infectious viral diseases. Nat Med 2021;27:401–10. 33723456 10.1038/s41591-021-01282-0

[63] Akimov Y , Aittokallio T. Re-defining synthetic lethality by phenotypic profiling for precision oncology. Cell Chem Biol 2021;28:246–56. 33631125 10.1016/j.chembiol.2021.01.026

[64] Navare AT , Mast FD, Olivier JP, et al. Viral protein engagement of GBF1 induces host cell vulnerability through synthetic lethality. bioRxiv 2020. 10.1101/2020.10.12.336487. PMC9623979 36305789

[65] Pal LR , Cheng K, Nair NU, et al. Synthetic lethality-based prediction of anti-SARS-CoV-2 targets. iScience 2022;25:104311. 35502318 10.1016/j.isci.2022.104311 PMC9044693

[66] Mast FD , Navare AT, van der Sloot AM, et al. Crippling life support for SARS-CoV-2 and other viruses through synthetic lethality. J Cell Biol 2020;219:e202006159. 32785687 10.1083/jcb.202006159 PMC7659715

[67] Smedley D , Köhler S, Czeschik JC, et al. Walking the interactome for candidate prioritization in exome sequencing studies of Mendelian diseases. Bioinformatics 2014;30:3215–22. 25078397 10.1093/bioinformatics/btu508 PMC4221119

[68] Wu T , Hu E, Xu S, et al. clusterProfiler 4.0: a universal enrichment tool for interpreting omics data. The Innovation 2021;2:100141. 34557778 10.1016/j.xinn.2021.100141 PMC8454663

[69] Carlson M , Falcon S, Pages H, et al. org. Hs. eg. db: genome wide annotation for human. *R package version*. 2019;3:3.

[70] Tran AN , Dussaq AM, Kennell T, et al. HPAanalyze: an R package that facilitates the retrieval and analysis of the human protein atlas data. BMC Bioinformatics 2019;20:1–11. 31500569 10.1186/s12859-019-3059-z PMC6734269

