# Supplementary material for: Discovery of host-directed modulators of virus infection by probing the SARS-CoV-2–host protein–protein interaction network
Source: Brief Bioinform. 2022 Oct 27;23(6):bbac456. doi: 10.1093/bib/bbac456 (PMC9677461; doi:10.1093/bib/bbac456)
Supplement: SUPPLEMENTARY_FIGURES_bbac456 [file supplementary_figures_bbac456.pdf]

Supplementary Figures for

Discovery of host-directed modulators of virus infection by probing the SARS-CoV-2-host protein-protein interaction network

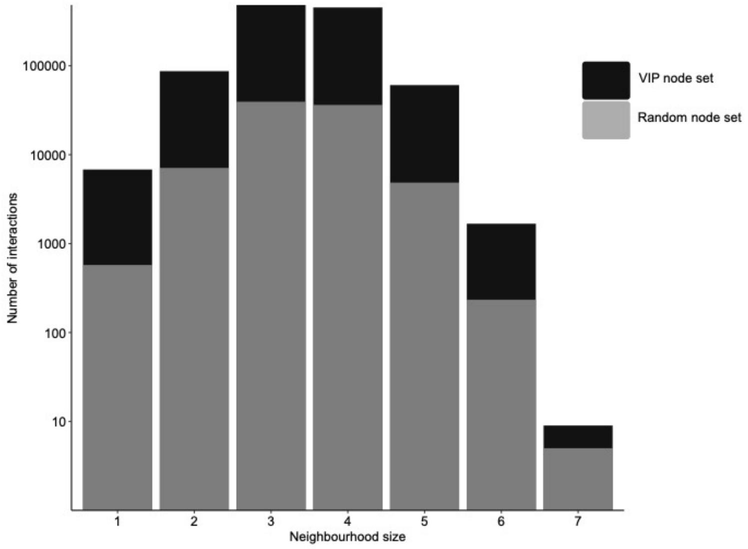

Figure S1. Neighbourhood (shortest path distance) from VIP to non-VIP nodes compared to a random node set of the same size as the number of VIPs in the PPI network (297 VIPs).

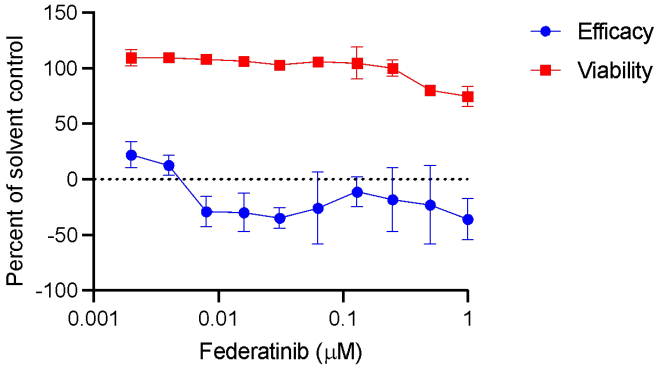

Figure S2. The effect of federatinib on SARS-CoV-2 infection. 293TAT cells were treated with the indicated concentrations of compounds immediately prior to infection with SARS-CoV-2 at a MOI of 0.01. Parallel wells contained cells treated only with compounds to study toxicity. Forty-eight hours post infection, cell viability was assessed using CellTiter-Glo and antiviral efficacy and viability were calculated as described in Materials and Methods. Data points reflect average and standard deviations of triplicate experiments per condition.

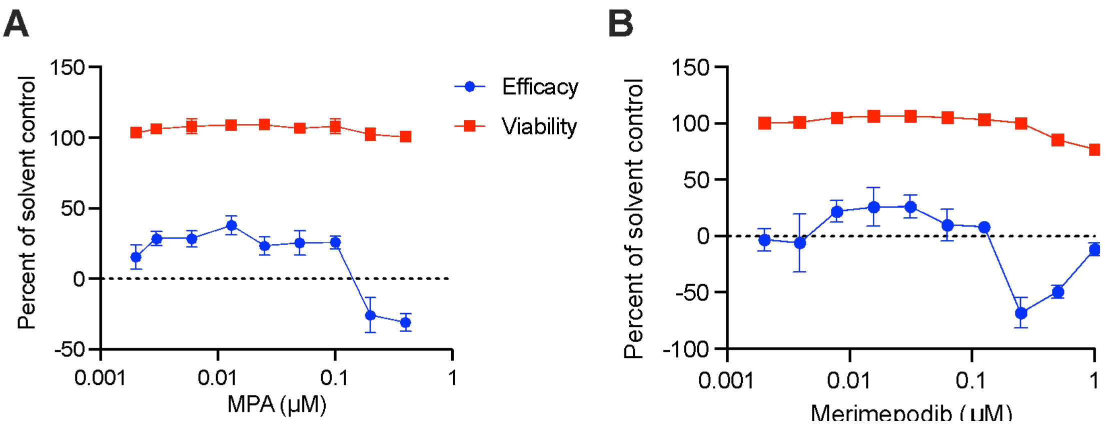

Figure S3. Effect of IMPDH inhibitors mycophenolic acid (MPA) and merimepodib on SARS-CoV-2 infection in 293TAT cells. MPA showed a maximum antiviral efficacy of 39% at 0.01 µM (Figure A), and merimepodib showed peak antiviral effect of 26% at 0.01-0.03 µM (Figure B). However, there was no dose dependency in the observed antiviral efficacy in 293TAT cells. 293TAT cells were treated with the indicated concentrations of compounds prior to infection with SARS-CoV-2 at a MOI of 0.01. Parallel wells contained cells treated only with compounds to study toxicity. Forty-eight hours post infection, cell viability was assessed using CellTiter-Glo and antiviral efficacy and viability were calculated as described in Materials and Methods. Data points reflect average and standard deviations of triplicate experiments per condition.

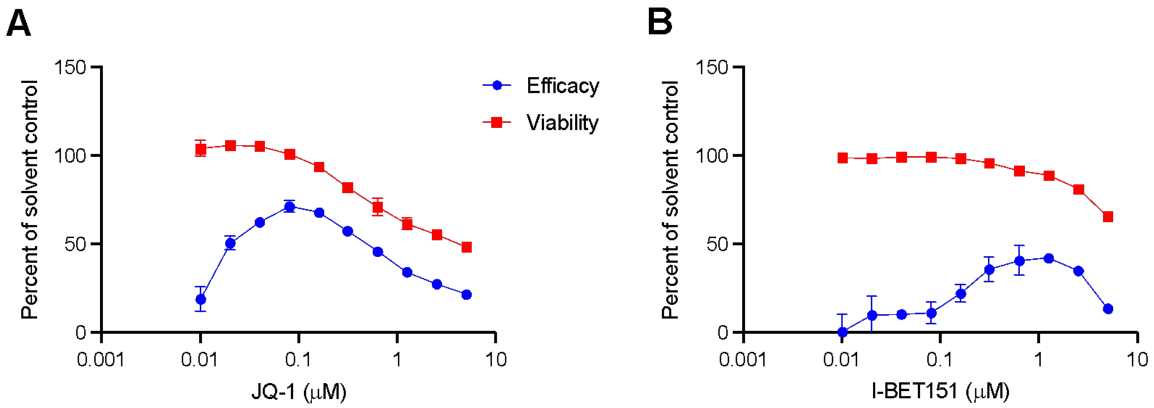

Figure S4. The effects of JQ1 and I-BET151 on SARS-CoV-2 infection. Calu-3 cells were treated with the indicated concentrations of compounds immediately prior to infection with SARS-CoV-2 at a MOI of 0.1. Parallel wells contained cells treated only with compounds to study toxicity. Ninety-six hours post infection, cell viability was assessed using CellTiter-Glo and antiviral efficacy and viability were calculated as described in the Materials and Methods. Data points reflect average and standard deviations of triplicate experiments per condition.

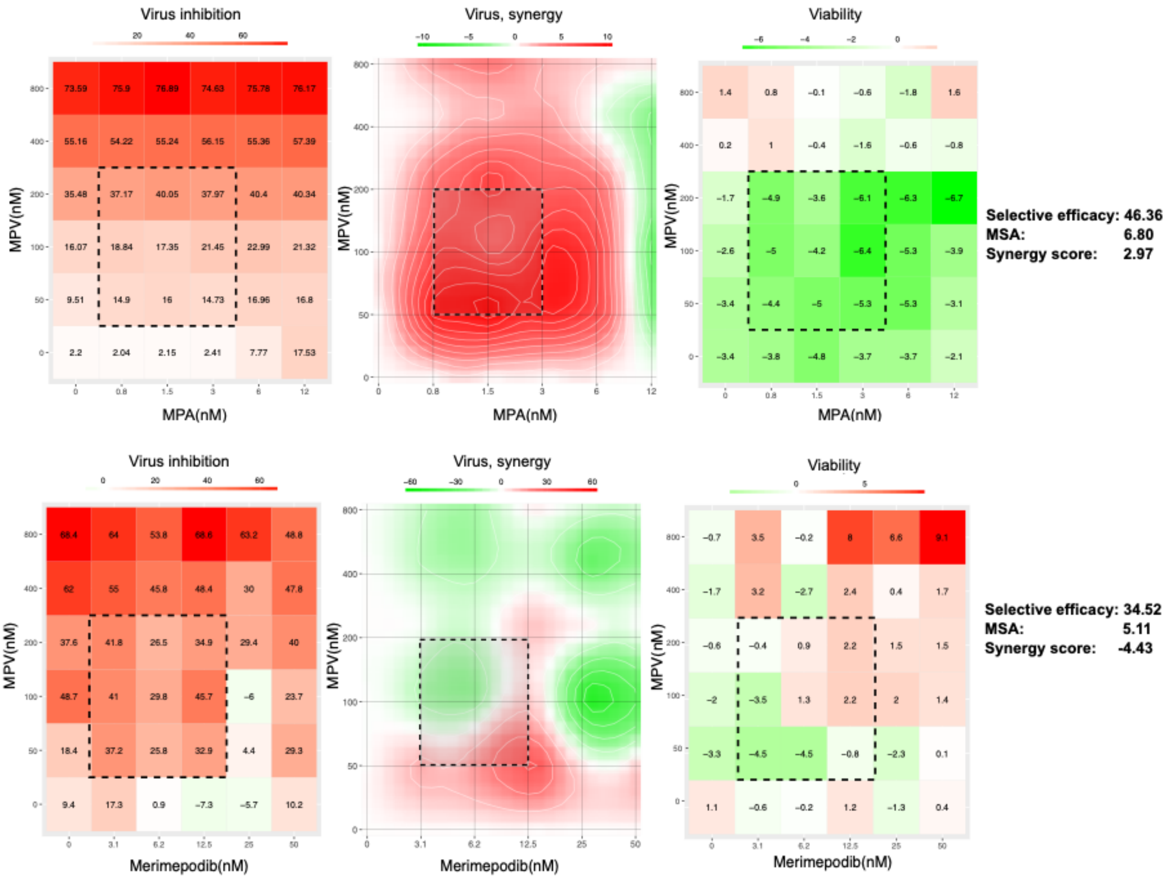

Figure S5. Dose-response matrix of SARS-CoV-2-infected 293TAT cells when combining molnupiravir (EIDD-1931) with either (A) MPA or (B) merimepodib. The maximum synergistic area (dotted-line square) and the Bliss synergy score were calculated with SynergyFinder v2.0, both for efficacy (virus infection) and viability (toxic effect). Selective efficacy quantifies the difference in inhibition of virus-infected and mock-infected cells. A selective efficacy of 100 means that the drug combination inhibits 100% of the virus-infected cells and does not affect the mock infected, drug-treated cells, while a selective efficacy of 0 means the drug kills both the virus and mock-infected cells. The selective efficacies of 46% and 35% for combinations of molnupiravir with MPA and merimepodib, respectively, indicate relatively high selective suppression of virus infection only. This is also seen in minimal co-inhibition of the non-infected cells.

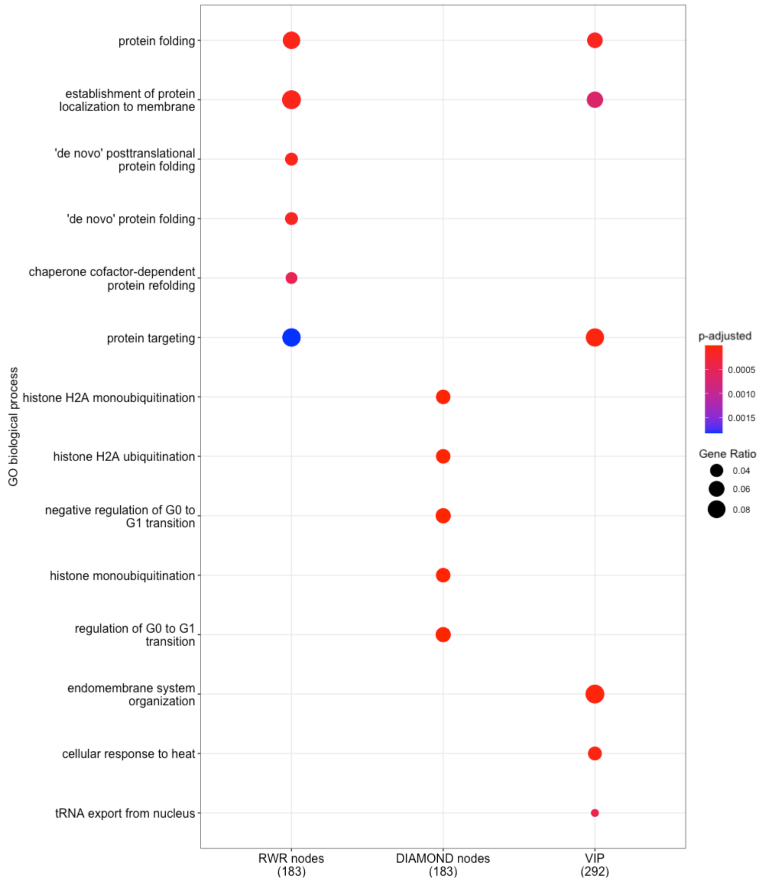

Figure S6. Comparison of the GO biological process between proteins identified by the RWR and DiAMoND algorithms and those enriched among the VIP nodes. The numbers in parentheses below each node set is the number of identified proteins in that set that have GO information available. The dots are colour-coded based on their corresponding adjusted p-values, and the dot sizes correspond to the gene ratio (i.e., genes of interest in the GO term/total number of genes of interest).
