# Supplementary material for: Discovery of host-directed modulators of virus infection by probing the SARS-CoV-2–host protein–protein interaction network
Source: Brief Bioinform. 2022 Oct 27;23(6):bbac456. doi: 10.1093/bib/bbac456 (PMC9677461; doi:10.1093/bib/bbac456)
Supplement: SUPPLEMENTARY_TEXT_bbac456 [file supplementary_text_bbac456.pdf]

Supplementary Text for

Discovery of host-directed modulators of virus infection by probing the SARS-CoV-2-host protein-protein interaction network

Vandana Ravindran, Jessica Wagoner, Paschalis Athanasiadis, Andreas B. Den Hartigh, Julia M. Sidorova, Aleksandr Ianevski, Susan L. Fink, Arnoldo Frigessi, Judith White, Stephen J. Polyak and Tero Aittokallio

Identification of host targets using network-based prioritization

We used a network-based protein prioritisation approach, the random walk with restart (RWR), to identify host protein targets closest to the VIP set. A random walk is a stochastic process, where the steps on the network take place with a certain probability. The process visits a sequence of adjacent nodes, forming a path, where at each step the next node is chosen at uniformly random among the neighbours of the current node. A RWR implements an additional restart probability, meaning that for every step taken in any direction, there is a probability of going back to the initial node. The advantage of the RWR method is that it provides a good representation of the topological similarity between the seed nodes and other nodes in the network.

In the RWR method, each protein node in the network is ranked in the descending order based on the probability of them being visited by a random walker that starts from any of the seed nodes (here, the VIP nodes). We implemented RWR using the Arete Cytoscape plugin that provides an exact solution of RWR, meaning it does not simulate a random walk, and therefore the method is much faster than the alternative algorithms available for calculating random walk [1,2].

The algorithm starts from a source node *s* and it is allowed to restart the walk in every time step at node *s* with a probability of *r.* Formally, the random walk with restart (RWR) is defined as

$$p_{t+1} = (1-r)Wp_t + rp_0 \qquad (1)$$

The transition matrix *W* is the column-normalised adjacency matrix of the graph and p_t is a vector that consists of the probabilities of being at node *i* at time step *t.* The initial probability vector p_0 is constructed such that the seed/source nodes (in this case nodes representing VIPs) are assigned equal probabilities. The candidate protein targets are then ranked according to the values of the steady-state probability vector p_∞.

Instead iteratively running the nonhomogeneous first-order matrix difference equation (1) until convergence, the Arete method determines the closed-form solution of the steady-state vector as follows:

$$p_\infty = r(I-(1-r)W)^{-1}p_0 \qquad (2)$$

The constant matrix $R = r(I - (1-r)W)^{-1}$ is pre calculated and the exact solution is obtained by matrix multiplication as $p_\infty = Rp_0$. This calculation is further simplified as many elements of the vector p_0 are zero, with only elements representing the VIP proteins having non-zero values. Since the system is solved exactly using the inverse of an invertible matrix and also the graph is connected in our case (we consider the giant component of the network), the linear difference equation (1) is stable and p_t converges asymptotically to the steady state p_∞. To guarantee the convergence, we ran the Arete method 15 times on our network and obtained always the same set of RWR nodes.

We compared the network-based RWR algorithm with DIAMOnD, an iterative, local neighbourhood-based node prioritisation method [3]. The DIAMonD algorithm starts by considering the immediate neighbours of the seed nodes (here, VIPs), and then selects a node with the smallest probability of having as many edges to the seed nodes as that of a randomly selected protein according to the hypergeometric distribution. After the next node is selected, it is added to the seed set, and the process is repeated until the desired number of candidate nodes have been selected and ranked in the ascending order based on the iteration step at which they were selected.

The parameter values for both of these algorithms (RWR and DIAMOnD) were set to their default parameters; the restart probability in RWR was set to 0.7, and the number of ranked proteins in DIAMOnD was set to 200, which was the recommended value, since at ~200 iterations, the number of the DIAMOnD-prioritised proteins with direct biological evidence was shown to reach a plateau, suggesting 200 as the maximal number that should be considered [3]. We therefore selected the top-200 ranked proteins also in the RWR algorithm to match the size of the protein set identified by DIAMOnD.

We observed that the proteins identified by the RWR algorithm were more enriched in similar biological pathways with the VIP proteins, when compared to those identified by the DIAMoND algorithm (**Figure S6**). Since the RWR-prioritized proteins were more related to the VIP nodes in terms of their biological functions, we considered the RWR identified host proteins for further analysis based on the top-200 nodes. The use of other cut-off values led to the same set of GO biological terms than those identified using the top-200 nodes, although their enrichment values differed slightly.

All the network visualisations were made using the Cytoscape software [4], and the network topological parameters, such as degree connectivity and betweenness centrality, were computed using the igraph R package [5].

Benchmarking network-based predictions using NCATS database

When comparing against the NCATS compound database [6], we converted the ChEMBL IDs into PubChem SIDs. The antiviral compounds were classified based on the curve rank, which is a numeric score, developed internally by NCATS, that combines classes of the dose-response curves and efficacy, such that more potent and effective compounds with higher quality curves are assigned a higher rank [6]. In our analyses, all compounds with a curve rank >0 were classified as antivirals [7].

We compared our protein identification method with another computational method that integrates three network-based drug repurposing algorithms that rely on artificial intelligence, network diffusion and network proximity [8]. In this work by Gysi et al., they experimentally tested in VeroE6 cell lines 918 compounds among the 6340 identified by their computational method. Out of these 918 compounds, only 8.38% (77/918) showed either weak or strong activity against SARS-CoV-2 (strong activity required efficacy over a broad range of concentrations). The remaining 91.6% (841/918) compounds showed no antiviral activity against SARS-COV-2. We further compared the 918 compounds with the NCATS database, and found that 73 compounds were common, out of which 21.9% (16/73) were validated as antivirals by the NCATS assay. Compared to the NACTS success rate of 7.7% (730/9479), the computational method proposed by Gysi et al. therefore led to 2.85-fold increase in the prediction of antiviral compounds.

We also compared the compounds identified by our network-based method against the experimental validations carried out by Gysi et al. [8]. In total, 70 compounds were common between our list and the 918 compounds verified by the authors in VeroE6 cell lines. Among these, 17.5% (12/70) were classified as antivirals by Gysi et al. in VeroE6 cell lines. We therefore obtained a 1.28-fold improvement in accuracy of identifying antiviral compounds, when compared to 8.38% (77/918) accuracy by their computational method. Even if these comparisons provide some kind of reference for the general accuracy of network-based prioritization methods, the number of overlapping compounds between the methods and screens remain quite limited, making the comparisons potentially biased. Our PPI network only considers interactions verified in 293T cell lines to create a more context specific network, which is expected to decrease the success rate when comparing to experimental validations performed in other cell types.

Statistical analysis

All statistical analyses were performed using R [9] and Python [10]. The two-sample Kolmogorov-Smirnov test was employed to compare the distributions of compounds and targets between the VIP and NIP sets, with the null hypothesis being that the distributions are similar, using the Scipy package [11].

Cell lines, culture condition and compounds

Calu-3 cells were maintained in standard medium (Minimum Essential Medium (MEM; catalogue number 11095; Gibco) supplemented with 9% foetal bovine serum (FBS; catalogue number SH3007103; HyClone) and 1% penicillin-streptomycin (catalogue number 15140; Gibco); 293TAT cells were maintained in DMEM (catalogue number 11995; Gibco), 9% FBS (catalogue number SH3007103; HyClone), 1% penicillin-streptomycin (catalogue number 15140; Gibco), 1% non-essential amino acids (NEAA; catalogue number 11140; Gibco), and 20mM HEPES (catalogue number 15630; Gibco). Compounds vorinostat (catalogue number S1047; Sellckchem), romidespin (catalogue number S3020; Sellckchem), spautin-1 (catalogue number S7888; Sellckchem), fedratinib (catalogue number S2736; Sellckchem), merimepodib (catalogue number S6689; Sellckchem), mycophenolic acid (catalogue number HYB0421; MedChem Express), (+)-JQ-1 (catalogue number HY13030; MedChem Express) and Molnupiravir (catalogue number HY125033 MedChem Express) were tested.

Antiviral efficacy was calculated by comparing the relative light units (RLU) from virus infected (IFX) cells treated with drugs (IFXdrug) compared to infected cells treated with solvent control (IFXDMSO), and non-infected cells (nonIFX) treated with solvent control (nonIFXDMSO). Cell viability was calculated by comparing the RLU from non-infected cells (nonIFX) treated with drugs (nonIFXdrug) with non-infected cells treated with solvent control (nonIFXDMSO) using the following equations:

$$Efficacy = [\frac{(IFX_{drug}) - (average(IFX_{DMSO})}{(average(nonIFX_{DMSO})) - (average(IFX_{DMSO})}] * 100$$

$$Viability = [\frac{nonIFX_{drug}}{average(nonIFX_{DMSO})}] * 100$$

SARS-CoV-2 qRT-PCR assay

RNA was isolated using the SingleShot Cell lysis kit (Bio-Rad). Briefly, at designated time points, cells were washed 1x with PBS followed by addition of a lysis buffer. After a 5-minute incubation at room temperature, liquid was transferred to a PCR plate and further incubated at 37C for 5 min followed by 5 min at 75C. The resulting RNA was used to synthesise complementary DNA (cDNA) using the iScript cDNA Synthesis kit (Bio-Rad). Briefly, 6 µL of RNA was mixed with 2 µL of reaction buffer, 1,5 µL of water, and 0.5 µL of reverse transcriptase. This was incubated for 30 min at 42C followed by 5 min at 85C. 10 µL of water was added to each product to dilute the cDNA. Quantitative RT-PCR was performed on a Roche Lightcycler 480 using SYBR Green (Bio-Rad) with the following primers (all primers listed in the 5' to 3' orientation):

human *RPS18*: TGC GAG TAC TCA ACA CCA ACA and CTT CGG CCC ACA CCC TTA AT

SARS-CoV-2 E: ACAGGTACGTTAATAGTTAATAGCGT and ATATTGCAGCAGTACGCACACA

For each reaction 2 µL of cDNA was mixed with 5 µL of SYBR, 2 µL of water and 0.5 µL of each primer. The RT-PCR reaction was 40 cycles of 5 seconds at 95C and 1 minute at 62C, followed by melt curve analysis. Melt curve analysis was used to assess whether single reaction products were produced. Expression was calculated relative to the housekeeping gene *RPS18*.

Drug combination analysis

The two-drug checkerboard assays were carried out as before [12]. The multi-dose combination experiment data were analysed in SynergyFinder v2.0 web-application (https://synergyfinder.fimm.fi), an open-source software for multi-drug combination analysis [13]. We used the Bliss independence model to quantify the combination synergy. This model is based on a stochastic process in which drugs elicit their effects independently, and based on the probability of independent events, the expected combination effect can be calculated [14]. The average Bliss synergy score over the entire dose-response matrix was calculated with SynergyFinder v2.0, along with the Maximum Synergistic Area (MSA), which is a 3 x 3

dose-response area of the matrix, where the drug combination shows the highest synergistic effect. The selective efficacy was calculated by subtracting the toxicity (viability of mock-infected control cells) from the efficacy (inhibition of virus-infected cells). The selective efficacy quantifies the difference between efficacy and toxicity, meaning that a selective efficacy of 100 indicates that the drug combination inhibits the virus 100% and does not affect the mock-infected cells, while a selective efficacy of 0 indicates that the drug inhibits 100% of both the virus and mock-infected cells, indicating a high toxicity. The outliers for MPA at doses 3,6 and 12nM were corrected using DECREASE web-application (https://decrease.fimm.fi/) [15].

References

1. Lysenko A, Boroevich KA, Tsunoda T. Arete–candidate gene prioritization using biological network topology with additional evidence types. BioData Min. 2017; 10:1–12
2. Smedley D, Köhler S, Czeschik JC, et al. Walking the interactome for candidate prioritization in exome sequencing studies of Mendelian diseases. Bioinformatics 2014; 30:3215–3222
3. Ghiassian SD, Menche J, Barabási A-L. A DIseAse MOdule Detection (DIAMOnD) Algorithm Derived from a Systematic Analysis of Connectivity Patterns of Disease Proteins in the Human Interactome. PLOS Comput. Biol. 2015; 11:e1004120
4. Shannon P, Markiel A, Ozier O, et al. Cytoscape: a software environment for integrated models of biomolecular interaction networks. Genome Res. 2003; 13:2498–2504
5. Csardi G, Nepusz T, others. The igraph software package for complex network research. InterJournal Complex Syst. 2006; 1695:1–9
6. Inglese J, Auld DS, Jadhav A, et al. Quantitative high-throughput screening: a titration-based approach that efficiently identifies biological activities in large chemical libraries. Proc. Natl. Acad. Sci. 2006; 103:11473–11478
7. Xu T, Zheng W, Huang R. High-throughput screening assays for SARS-CoV-2 drug development: current status and future directions. Drug Discov. Today 2021; 26:2439–2444
8. Morselli Gysi D, do Valle Í, Zitnik M, et al. Network medicine framework for identifying drug-repurposing opportunities for COVID-19. Proc. Natl. Acad. Sci. 2021; 118:e2025581118
9. Team RC. R Core Team R: a language and environment for statistical computing. Found. Stat. Comput. 2020;
10. Van Rossum G, Drake FL. Python/C Api Manual-Python 3. 2009;
11. Virtanen P, Gommers R, Oliphant TE, et al. SciPy 1.0: fundamental algorithms for scientific computing in Python. Nat. Methods 2020; 17:261–272
12. Herring S, Oda JM, Wagoner J, et al. Inhibition of arenaviruses by combinations of orally available approved drugs. Antimicrob. Agents Chemother. 2021; 65:e01146-20
13. Ianevski A, Giri AK, Aittokallio T. SynergyFinder 2.0: visual analytics of multi-drug combination synergies. Nucleic Acids Res. 2020; 48:W488–W493
14. Bliss CI. The toxicity of poisons applied jointly. Ann. Appl. Biol. 1939; 26:585–615
15. Ianevski A, Giri AK, Gautam P, et al. Prediction of drug combination effects with a minimal set of experiments. Nat. Mach. Intell. 2019; 1:568–577
